# Advanced Therapy Medicinal Products for the Eye: Definitions and Regulatory Framework

**DOI:** 10.3390/pharmaceutics13030347

**Published:** 2021-03-06

**Authors:** Marina López-Paniagua, Ana de la Mata, Sara Galindo, Francisco Blázquez, Margarita Calonge, Teresa Nieto-Miguel

**Affiliations:** 1 Ocular Surface Group, Instituto de Oftalmobiología Aplicada (IOBA), University of Valladolid, Campus Miguel Delibes, Paseo de Belén 17, 47011 Valladolid, Spain; marina@ioba.med.uva.es (M.L.-P.); adelamatas@ioba.med.uva.es (A.d.l.M.); sgalindor@ioba.med.uva.es (S.G.); calonge@ioba.med.uva.es (M.C.); 2 Centro de Investigación Biomédica en Red de Bioingeniería, Biomateriales y Nanomedicina (CIBER-BBN), Instituto de Salud Carlos III, 28029 Madrid, Spain; 3 Centro en Red de Medicina Regenerativa y Terapia Celular de Castilla y León, 47007 Valladolid, Spain; 4 Clinical Trials Unit, Instituto de Oftalmobiología Aplicada (IOBA), University of Valladolid, Campus Miguel Delibes, Paseo de Belén 17, 47011 Valladolid, Spain; blazquez@ioba.med.uva.es

**Keywords:** advanced therapy medicinal product, ATMP, cell therapy, tissue engineering, gene therapy, eye, ocular, ophthalmology, regulatory, marketing authorization

## Abstract

Advanced therapy medicinal products (ATMPs) are a group of innovative and complex biological products for human use that comprises somatic cell therapy medicinal products, tissue engineered products, gene therapy medicinal products, and the so-called combined ATMPs that consist of one of the previous three categories combined with one or more medical devices. During the last few years, the development of ATMPs for the treatment of eye diseases has become a fast-growing field as it offers the potential to find novel therapeutic approaches for treating pathologies that today have no cure or are just subjected to symptomatic treatments. Therefore, it is important for all professionals working in this field to be familiar with the regulatory principles associated with these types of innovative products. In this review, we outline the legal framework that regulates the development of ATMPs in the European Union and other international jurisdictions, and the criteria that each type of ATMP must meet to be classified as such. To illustrate each legal definition, ATMPs that have already completed the research and development stages and that are currently used for the treatment of eye diseases are presented as examples.

## 1. Introduction

Advanced therapy medicinal products (ATMPs) are a large and diverse group of therapeutic agents for human use. They consist of somatic cell therapy medicinal products (sCTMPs), tissue engineered products (TEPs), gene therapy medicinal products (GTMPs), and the so-called combined ATMPs (cATMPs) that include one of the previous three categories combined with one or more medical devices as an integral part of the product [1].

As with other existing and often less-complex medicinal products, ATMPs must meet the same high standards for scientific, methodological, and regulatory requirements: (1) the safety and efficacy must be demonstrated through both preclinical studies and human clinical trials. Human trials must be designed and conducted following European Union (EU) regulation No. 536/2014 and comply with the principles of good clinical practice (GCP) as stated in Commission Directive 2005/28/EC [2,3]; (2) production must comply with the principles of good manufacturing practices (GMP) [4]; and (3) standard post-authorization and pharmacovigilance requirements must be met [5]. Unlike other more common drugs, ATMPs are medicinal products with a very high degree of complexity that are associated with not only their composition, but also with all of the processes that are necessary for proper development, i.e., manufacturing, characterization, and marketing authorization. Due to the complex nature of ATMPs, they are generating great scientific, clinical, and regulatory challenges for all linked professionals, including researchers, clinicians, developers, and regulators [6]. 

In the EU, the legal framework for ATMPs is regulated by the European Medicines Agency (EMA), established to guarantee that all products classified as ATMPs were subjected to the proper regulatory assessment prior to clinical and commercial use [1]. A key point in the introduction of ATMP regulation was the establishment of the Committee for Advanced Therapies (CAT) in 2009. The CAT is a multidisciplinary body within the EMA that is responsible for ATMP classification; assessment of quality, safety, and efficacy; performing primary evaluation of marketing authorization applications; and monitoring all of the scientific advancements of the field [7].

The field of ATMPs is currently at the forefront of innovation as it offers novel therapeutic approaches for the treatment of pathologies that, at present, have limited or no effective alternatives. ATMPs hold the potential of curing or preventing the progression of a wide variety of severe and incapacitating diseases, such as some types of cancer, Alzheimer’s disease, Parkinson’s disease, etc., that today are untreatable or are just subject to palliative treatments [8]. For several reasons, the eye is an ideal organ for application of ATMPs. First, it has small dimensions, thus requiring low amounts of medicinal product for treatment. Second, the anatomical structure is compartmentalized, thus limiting the distribution of medicinal product to non-target tissues. Third, it has good accessibility for applying treatments and examining outcomes. Fourth, it is isolated from the rest of the body due to the blood–retinal barrier. This makes the eyeball an immunologically privileged site, because it restricts the passage of immunoglobulins. These reasons are why ATMPs present a great potential to improve the prognosis and potentially cure ocular diseases that currently have no effective treatment such as age-related macular degeneration, retinitis pigmentosa, Leber’s congenital amaurosis, Stargardt’s disease, optic nerve pathology, and limbal stem cell deficiency, among others.

During the last few years, the development of ATMPs for the treatment of eye diseases has become a fast-growing field. Therefore, it is important for all professionals working in this field to be familiarized with the regulatory principles associated with these types of innovative products. The aim of this review is to present the legal framework that regulates the development of ATMPs in the EU and the criteria that each type of ATMP must meet to be classified as such. We also identify and describe the ATMPs for the eye that have completed the research and development stages and are currently being used for the treatment of ocular diseases.

## 2. ATMP Regulatory Framework

### 2.1. ATMP Regulatory Framework in the EU

In the EU, the legal framework that regulates all medicinal products for human use, among which are ATMPs, is principally established in Directive 2001/83/EC [9]. The EMA is responsible for implementing this framework in cooperation with the national regulatory agencies from each EU member state. Directive 2001/83/EC defines a medicinal product as follows: “(1) any substance or combination of substances presented as having properties for treating or preventing disease in human beings; or (2) any substance or combination of substances which may be used in or administered to human beings, either with a view to restoring, correcting or modifying physiological functions by exerting a pharmacological, immunological, or metabolic action or to making a medical diagnosis”.

ATMPs are medicinal products that include engineered cells and/or tissues or recombinant nucleic acids; therefore, they are under the regulatory framework of biological products. The specific legal framework for ATMPs was established by the European Commission in Regulation EC No. 1394/2007, and it provides the regulatory principles for the evaluation, authorization, and post-authorization follow-up for ATMPs that are intended to be commercialized in any EU member state (Table 1). 

Under the EU regulatory framework, it is compulsory to get a marketing authorization prior to commercializing any medicinal product in any EU member state. As with all medicinal products, to get marketing authorization for an ATMP, it is necessary that the manufacture of the product be performed in compliance with the guidelines for GMPs described in Commission Directive 2003/94/EC [4]. Furthermore, following regulation (EU) No. 536/2014 [2], the product must undergo clinical trials to demonstrate that it is safe and effective in patients. ATMP clinical trial authorization depends on the national competent authorities where the trial will be performed. However, all ATMP marketing authorization applications are evaluated via the EMA’s centralized procedure to guarantee that they follow a single evaluation, and get an authorization that is valid throughout the EU [10,11]. 

**Figure 1:**
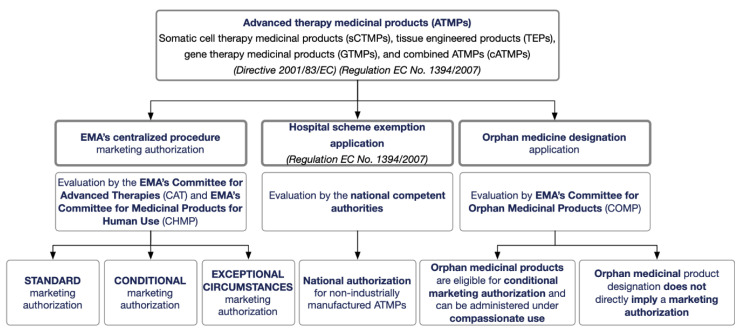
Regulatory pathway for advanced therapy medicinal products (ATMPs) in the European Union (EU). The European Medicines Agency (EMA) is responsible for implementing this regulatory framework in cooperation with the national regulatory agencies from each EU member state.

EMA’s centralized procedure can grant three different types of marketing authorization: standard marketing authorization, conditional marketing authorization, and marketing authorization under exceptional circumstances (Figure 1). The type of marketing authorization requested will depend on whether or not the ATMP meets an unmet medical need and/or on the demonstration of a positive benefit-risk balance provided by enough scientific and medical data obtained during development [12]. Nevertheless, under ATMP Regulation EC No. 1394/2007, the so-called “hospital scheme exemption” opens the possibility for a national authorization of non-industrially manufactured ATMP, i.e., a custom-made product designed and produced for an individual patient. Such an ATMP can be used on a non-routine basis within the same member state in a hospital under the exclusive responsibility of a specific medical practitioner (Figure 1) [1].

As described above, the CAT plays a key role in the regulatory oversight of ATMPs. This committee of experts in both the scientific and regulatory aspects of ATMPs is responsible for the primary evaluation of ATMP marketing authorization applications for EMA’s Committee for Medicinal Products for Human Use. One of its duties is to provide scientific recommendations for the classification of ATMPs [1,13]. To determine if a putative gene- or cell/tissue-based product fulfills the criteria to be considered as an ATMP, developers can apply for the ATMP classification procedure provided by the CAT. The main purpose of this procedure is to help developers evaluate cases where the classification of a product is not clear. Within 60 days upon receiving the application, the CAT should give its recommendations based on the information supplied by the developer. In this way, the CAT provides assistance regarding the regulatory and development path that should be followed [14]. In the case of cATMPs, the CAT works together with the national regulatory authorities in charge of medical devices of each EU member state with the aim of providing joint recommendations [15].

An ATMP can also be designated as an orphan medicinal product by the Committee for Orphan Medicinal Products (COMP) of the EMA. Orphan designation depends on three criteria: (1) it diagnoses, prevents, or treats a life-threatening or chronically debilitating disease; (2) the disease affects no more than 5 in 10,000 people in the EU or has insufficient returns on investment; and (3) there is a lack of alternative methods of diagnosis, prevention, or treatment [16]. Orphan medicine designation does not directly imply a marketing authorization because demonstration of quality, safety, and efficacy are not preceding requirements. However, designated orphan medicines are eligible for conditional marketing authorization, allowing administration of an unauthorized medicine to patients under compassionate use outside a clinical study. In addition, orphan medicinal products can benefit from incentives such as protection from competition once on the market (Figure 1).

### 2.2. Regulatory Framework for Cell- and Gene-Based Therapies in Other Jurisdictions

Although the term ATMP is specific for cell- and gene-based therapies developed for commercial use in the EU, other countries such as the United States (US), Japan, Canada, Australia, and Korea also have specific regulatory frameworks for these types of therapies [17]. Despite their differences, the regulatory frameworks of all jurisdictions share the same main goals, i.e., to guarantee the safety and rights of patients and to assure the quality of the results obtained from the preclinical and clinical studies that evaluate the safety and the efficacy of the therapies [17].

Great efforts are being made to achieve international harmonization of the regulatory frameworks for the development of medicinal products. The EU, US, and Japan are the founding members of the International Council for Harmonization of Technical Requirements for Pharmaceuticals for Human Use (ICH). The goal of this international council is to develop and establish worldwide adoption of the scientific, technical, and regulatory requirements for the development of human medicinal products. Therefore, the regulatory frameworks of these jurisdictions have great influence on the international development of specific cell- and gene-based therapies.

In the US, similar to the EU, cell- and gene-based therapies are regulated by the Food and Drug Administration (FDA) as a subset of biological medicinal products known as cellular and gene therapy products (Table 1). Although the inclusion criteria for defining a gene therapy product are similar to the ones in the EU regulatory framework, there is a difference in the criteria for classifying cell and tissue-based products. In both jurisdictions, to classify a cell- or a tissue-based product as an advanced therapy, the processing of the cells must include a manipulation that alters the native biological features; however, in the US, the term “manipulation” distinguishes between structural and non-structural cells and tissues (see Section 3.1) [18,19,20].

In Japan, the protection of public health safety is the responsibility of the Japanese Ministry of Health, Labour, and Welfare (MHLW) that works with the Pharmaceutical and Medical Devices Agency (PMDA). The PMDA is the regulatory authority responsible for ensuring the safety, efficacy, and quality of medical devices and pharmaceuticals (including biological products). Gene-, cell-, and tissue-based therapies are regulated under a special framework for regenerative medicine products by the Office of Cellular and Tissue-based (Table 1). Under the Japanese Pharmaceuticals and Medical Devices Act, regenerative medicine products are those that consist of processed human/animal cells that are designed to be used for reconstructing, repairing, or substituting human tissues or organs, or for treating or preventing human diseases. Products that contain modified cells with recombinant nucleic acids that are intended to be used for the treatment of human diseases are also considered regenerative medicine products [21,22].

## 3. Definitions and ATMP Classification Criteria

Legal definitions of ATMPs are essential because they facilitate the classification of a product and therefore determine its whole development plan according to the regulatory framework of each jurisdiction and/or region. Performing a correct classification at an early stage of development is a key step because it determines the itinerary to be followed in research and in preclinical and clinical studies.

In this review, we outline the legal definitions of each type of ATMP according to the EU regulatory framework and the criteria that they should meet to be classified as such. To illustrate each legal definition, we have selected as examples ATMPs that have completed the research and development stages in different jurisdictions and that are currently being used for the treatment of eye diseases.

### 3.1. Cell-Based Medicinal Products: Somatic Cell Therapy and Tissue-Engineered Medicinal Products

Cell-based or stem cell-based medicinal products encompass two types of therapies, sCTMPs and TEPs. The European Commission, through Regulation EC No. 1394/2007 and Directive 2001/83/EC, has provided precise legal definitions of both. However, due to the complex nature of these medicinal products and the rapid evolution of science in this field, questions about ATMP classification can emerge. This is especially so regarding the classification of an ATMP as sCTMP or TEP because both products include cells. Here, we review the legal definitions for cell-based medicinal products and the main points to be taken into consideration to classify an ATMP as sCTMP or TEP. In addition, we review cell-based therapies already authorized for ocular indication. 

The definition of sCTMP is currently included in Directive 2009/120/EC amending Directive 2001/83/EC of the European Parliament and of the Council on the European Community. An sCTMP is “a biological medicinal product whose active substance is made by a living organism” [23]. The product “has the following characteristics: (1) contains or consists of cells or tissues that have been subject to substantial manipulation so that biological characteristics, physiological functions or structural properties relevant for the intended clinical use have been altered, or of cells or tissues that are not intended to be used for the same essential function(s) in the recipient and the donor; (2) is presented as having properties for, or is used in or administered to human beings with a view to treating, preventing or diagnosing a disease through the pharmacological, immunological or metabolic action of its cells or tissues” [11]. For example, in vitro cultivation of cells or genetic modification of cells are considered substantial manipulations [14]. However, the following “shall not be considered as substantial manipulations: cutting, grinding, shaping, centrifugation, soaking in antibiotic or antimicrobial solutions, sterilization, irradiation, cell separation, concentration or purification, filtering, lyophilization, freezing, cryopreservation or vitrification” [1].

The definition of a TEP is provided by Regulation EC No. 1394/2007, where it is defined as “a product that contains or consists of engineered cells or tissues, and is presented as having properties for, or is used in or administered to human beings with a view to regenerate, repair or replace a human tissue” [1]. “Cells or tissues shall be considered ‘engineered’ if they fulfill at least one of the following conditions: (1) the cells or tissues have been subjected to substantial manipulation or (2) the cells or tissues are not intended to be used for the same essential function or functions in the recipient as in the donor” [1]. “A TEP may contain cells or tissues of human or animal origin, or both. The cells or tissues may be viable or non-viable.” [1] However, “products containing or consisting exclusively of non-viable human or animal cells and/or tissues, which do not contain any viable cells or tissues and which do not act principally by pharmacological, immunological or metabolic action, shall be excluded from this definition” [1]. “TEPs may also contain additional substances, such as cellular products, bio-molecules, biomaterials, chemical substances, scaffolds or matrices” [1].

**Figure 2:**
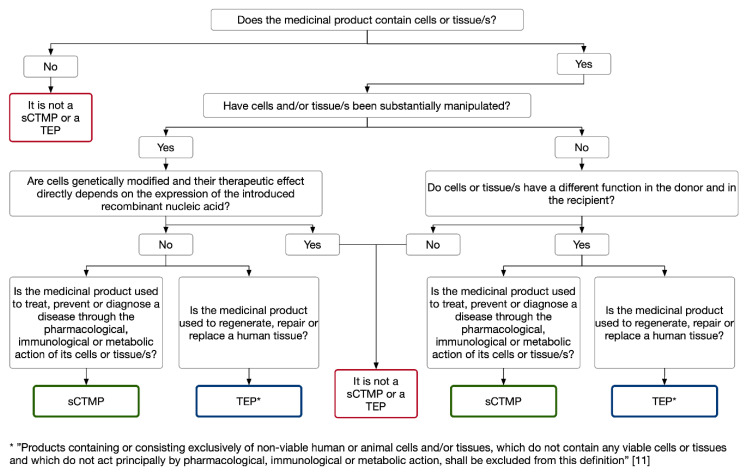
Easy guide to classify a cell-based medicinal product as a sCTMP or a TEP. Abbreviations: sCTMPs, somatic cell therapy medicinal products; TEPs, tissue engineered products. Modified from [14].

The main difference between sCTMPs and TEPs lies in the therapeutic action of these medicinal products. The sCTMPs are intended for treating, preventing, or diagnosing a disease through its pharmacological, immunological, or metabolic action. In contrast, TEPs are administered for regenerating, repairing, or replacing a human tissue. Therefore, when a researcher or a developer has doubts about whether an ATMP must be classified as a sCTMP or a TEP, the decision-making should be performed based on the mode of action of the ATMP (Figure 2) [14]. It should be considered that when “a product contains viable cells or tissues, the pharmacological, immunological or metabolic action of those cells or tissues shall be considered as the principal mode of action of the product” [1]. In addition, it is necessary to consider that it is possible that a cell-based medicinal product falls within the definition of both sCTMP and TEP. In this case, the medicinal product shall be considered as a TEP [1]. Nevertheless, when developers have doubts in determining if their ATMPs are sCTMPs or TEPs, they can apply for the ATMP classification procedure administered by the CAT and follow its recommendations.

Another critical situation related to the classification of the ATMP occurs when cells are modified by adding a mRNA sequence, and the therapeutic effect of the medicinal product depends directly on the protein encoded by the added mRNA. Here, it would be possible to argue the classification of the medicinal product as a GTMP. However, due to the short half-life of mRNA in the modified cells, probably little or no residual mRNA will remain inside the cells administered to the patients. Therefore, the recombinant nucleic acid is not administered to the recipient, and the medicinal therapy is not considered to comply with the definition of a gene therapy (see Section 2.2). This ATMP can be considered to be a sCTMP or TEP, depending on the function of the transplanted cells (with altered phenotype but no altered genotype) to the patient [14]. When an ATMP can fall within the definition of either a GTMP, sCTMP, or TEP, it is considered to be a GTMP [1].

In the US, ATMPs are regulated as biological products by the FDA. Here, cell- and tissue-based products are considered as biological drugs when they are subjected to more than minimal manipulation or non-homologous use. A minimal manipulation, in case of structural tissues, is defined as “processing that does not alter the original relevant characteristics of the tissue relating to the tissue’s utility for reconstruction, repair, or replacement”, and it is further defined as “processing that does not alter the relevant biological characteristics of cells or tissues” in the case of cells or non-structural tissues [20]. Moreover, there is not a sub-classification for cell-based biological products because all of them are included in the category of cellular therapy products [20]. In Japan, cell-based products are classified as regenerative medicinal products by the PMDA and are defined as processed human cells used to reconstruct, repair, or reform the physical structure of a human, or to treat or prevent disease. In this jurisdiction, “processing” is defined as “the artificial expansion/differentiation of cells, establishment of a cell line, chemical treatment to activate cells or tissues, modification of biological characteristics, combination with non-cell/tissue components, and genetic modification of cells, cells for non-homologous use” [22,24].

#### Somatic Cell Therapy and Tissue Engineered Medicinal Products for the Eye

**Figure 3:**
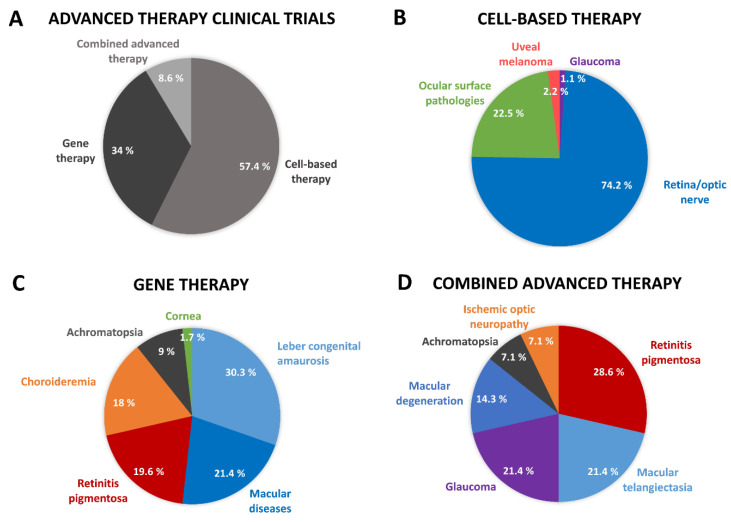
Clinical trials in which the safety and/or efficacy of advanced therapies for eye diseases are evaluated: (**A**) Advanced therapy clinical trials; (**B**) Cell-based therapy clinical trials; (**C**) Gene therapy clinical trials; (**D**) Combined advanced therapy clinical trials.

Currently, a high percentage of the clinical trials that are being carried out to study the efficacy and/or safety of ATMPs for eye diseases are focused on the analysis of cell-based medicinal products (Figure 3A). Four years ago, this percentage was around 76%, with two-thirds focused on sCTMPs and one-third on TEPs [8]. At the moment, there are several ongoing clinical trials in which efficacy and/or safety of cell-based medicinal products are being tested to treat, regenerate, repair, or replace human ocular tissues in diseases such as limbal stem cell deficiency, presbyopia, cataract, Stargardt’s macular dystrophy, acute non-arteritic anterior ischemic optic neuropathy, and retinitis pigmentosa [25]. In a recent search for the terms “cell therapy or tissue engineering” and “eye diseases” in the US National Library of Medicine (ClinicalTrials.gov, accessed on 4 February 2021), there were 607 clinical trials, but actually only 93 of them were performed to evaluate cell-based therapies. Of these 93 clinical trials, 22.5% (21 out of 93) had already been completed. Most of them (74.2%; 69 out of 93) were associated with retinal or optic nerve diseases, while another important proportion (22.5%; 21 out of 93) were associated with ocular surface pathologies. Only one out of 93 (1.1%) clinical trials was associated with glaucoma, and two were associated with uveal melanoma (2.2%) (Figure 3B).

The number of authorized sCMTPs or TEPs is not very large (Table 2), probably due to the technical difficulty and the high costs involved in developing a cell-based therapy and proving its safety and efficacy.

Nevertheless, several cell-based therapies have been authorized in the EU under the orphan medicinal product designation (Table 2). In June 2011, orphan medicinal product designation (EU/3/11/874) was approved by the EMA to a company in the United Kingdom (TMC Pharma Services Ltd., Hampshire, United Kingdom) for “human embryonic stem-cell-derived retinal pigment epithelial cells for the treatment of Stargardt’s disease”, a genetic disorder that affects retinal pigment epithelial cells and that leads to gradual loss of vision [26]. The human embryonic stem-cell-derived retinal pigment epithelial cells are administered to the patient by injection directly into the eye, under the retina, and are expected to help its function. Before its authorization in the EU, this medicinal product was granted approval in the US for the treatment of Stargardt’s macular dystrophy [27]. In December 2016, the sponsorship for this cell-based therapy was transferred to Astellas Pharma Europe B.V., The Netherlands.

In July 2013, orphan medicinal product designation (EU/3/13/1168) was approved by the European Commission to the University of Newcastle, United Kingdom, for “ex vivo expanded autologous human corneolimbal epithelium, containing stem cells, for the treatment of limbal stem cell deficiency”. Limbal stem cell deficiency is a pathology resulting from a critical reduction and/or dysfunction of the limbal epithelial stem cells that are responsible for the continuous renewal of the corneal epithelium. This pathology often results in corneal opacity, loss of vision, and/or chronic pain [28]. In this case, autologous human corneolimbal epithelial cells are cultured in vitro using a culture system that includes cells derived from the human placenta. Finally, with the expectation that the implanted stem cells will help the cornea to regenerate, the cultured corneolimbal epithelial cells are implanted on the damaged ocular surface [29]. In October 2014, orphan medicinal product designation (EU/3/14/1340) was awarded by the European Commission to NHS National Services Scotland (trading as Scottish National Blood Transfusion Service, United Kingdom), for “cultured allogeneic corneolimbal stem cells” to be used in the treatment of the same disease. Here, limbal stem cells are obtained from a donor eye and cultivated on a membrane. The membrane with the cultured cells is implanted onto the ocular surface of the patient, with the expectation that it will help corneal regeneration [30].

Another cell-based therapy, OraNera (CellSeed Europe Ltd.), to treat limbal stem cell deficiency in adults, was evaluated by the EMA (Table 2). In this case, the active substance was composed of autologous oral mucosal epithelial cells. In March 2013, after an application for a pediatric investigation plan, the company CellSeed Europe Ltd. informed the EMA that it wished to remove its application for a marketing authorization for OraNera. The basis for the removal request was the negative benefit–risk balance established by the CAT to use this medicinal product in patients with limbal stem cell deficiency [31].

The first ATMP approved, with marketing authorization, for ocular treatment in the EU was Holoclar, a cell-based therapy, specifically a TEP, to replace damaged ocular surface epithelium in patients suffering limbal stem cell deficiency. In 1997, Pellegrini et al. reported the first successful clinical trial, in which autologous epithelium from a limbal biopsy was cultured in vitro on petrolatum gauze or on a soft contact lens. It was then transplanted into two patients with limbal stem cell deficiency. After several studies that showed the presence of limbal epithelial stem cells in these types of cultures [32,33], and the selection of fibrin as a more suitable substratum for limbal epithelial cell cultivation [34], the product became a “routine treatment” in Italy in 2004 and was accepted in India some years after. However, this treatment was not established in the US because the regulatory requirements were not achieved [35]. In November 2008, orphan designation was approved by the European Commission to the Chiesi Farmaceutici S.P.A. (Italy), for “ex vitro expanded autologous human corneal epithelium containing stem cells for the treatment of corneal lesions, with associated corneal (limbal) stem cell deficiency, due to ocular burns” [36]. Finally, in February 2015, Holoclar, while the orphan designation, became the first stem cell-based ATMP to be approved with marketing authorization by the EMA [37], and given a conditional marketing authorization. This means that more clinical evidence on the safety and efficacy of this cell-based medicinal product must be collected and reported to the EMA to get the standard marketing authorization [38]. Currently, Holoclar is manufactured by Holostem Terapie Avanzate S.R.L. (Italy), which received the ownership of this product from Chiesi Farmaceutici S.P.A. in June 2020 [36].

Currently there are no FDA-authorized cellular therapy products for ophthalmic indications in the US [19]. In Japan, the PMDA has so far approved only one cell-based regenerative medicinal product to treat ocular diseases. In March 2020, the human somatic cell-processed orphan product Nepic (Japan Tissue Engineering Co., Ltd., Gamagori, Japan) was authorized. The active substance is a human autologous corneal-derived epithelial cell sheet to treat limbal stem cell deficiency (Table 2) [39]. 

### 3.2. Gene Therapy Medicinal Products

According to the EU, part IV of Annex I to Directive 2001/83/EC and the update of Directive 2009/120/EC, “a GTMP is defined as a biological medicinal product that presents the following two characteristics: (1) it contains and active substance that contains or consist of a recombinant nucleic acid used in or administered to human beings with a view to regulating, repairing, adding or deleting a genetic sequence; (2) its therapeutic, prophylactic or diagnostic effect relates directly to the recombinant nucleic acid sequence it contains, or to the product of genetic expression of this sequence. Moreover, GTMPs shall not include vaccines against infectious diseases” [1,9].

The obtention of these products involves the generation and amplification of genetic constructs in cell lines. The most commonly used technology for gene transfer is based on viral vectors, although non-viral vectors are also used, as they can be assembled synthetically. Further, the constructs are either purified for direct administration (non-cell-based), in vivo gene therapies, or used for the transduction of therapeutic cells (cell-based, or ex vivo, gene therapies) [12]. 

The goal of GTMPs is to deliver a gene with the intention to obtain, through its expression, a therapeutic effect in a patient. This gene should encode a protein that replaces the dysfunctional or absent protein in the patient, or a protein that inhibits the function related to the respective pathology [6]. A GTMP usually consists of a vector including the inserted sequence and the target cells that are modified by the vector, which finally encodes a protein and is expressed if the gene transfer is successful. 

Considering part IV of Annex I to Directive 2001/83/EC, there are some specific requirements for GTMPs: “(1) GTMP containing recombinant nucleic acid sequences or genetically modified microorganisms or virus, should contain an active substance consist of nucleic acid sequences or genetically modified microorganisms or viruses in its carrier for medical use. The product could also be combined with medical devices. (2) Regarding GTMP containing genetically modified cells, the finished medicinal product shall comprise genetically modified cells formulated in the final container for the proposed medical application. The final product could be also combined with a medical device” [9,11]. 

One important challenge of these products is to achieve a stable gene expression. The duration of the product depends on the promoter used to drive the transgene, the cell survival, the persistence of the transgene, and the immune response against the vector or the genetically modified cells. Another challenge of GTMPs is related to the clinical efficacy and safety. These depend on the gene transfer efficiency, the capacity of directing the vector to the target cells, and the expression level of the gene of interest. In parallel, the target cell type, the type of vector, and the administration are also important factors to be considered [40].

#### Gene Therapy Medicinal Products for the Eye

At present, a large number of gene therapy clinical trials, around 2700 performed in 38 different countries, have been approved since 1989. Most of them have addressed cancer (66.6%), while only 1.3% have been directed towards ocular diseases [41]. Nevertheless, among the organs targeted by gene therapy, the eye is at the vanguard of translational gene therapy largely due to appropriate disease targets and its suitable anatomic features: it presents a well-defined anatomy, it is relatively immune-privileged, it is easy to access and examine, and it is possible to use one eye as an experimental target and the other one as a control in the same subject [42]. 

Gene therapy could offer an improvement in the treatment of several ocular diseases like glaucoma, X-linked retinoschisis, Stargardt’s disease, choroideremia, retinitis pigmentosa, age-related macular degeneration (AMD), and Leber’s congenital amaurosis [42], among others. Some corneal diseases are also potentially open to gene therapy, including the monogenic lysosomal storage disorders like mucopolysaccharidosis type IV and VII, corneal scarring, corneal neovascularization, anterior and stromal dystrophies linked to genetic mutations, corneal graft rejection, and the maintenance of corneal endothelial cell density [43]. 

Currently, 56 clinical trials related to gene therapy for eye diseases are reported (ClinicalTrials.gov, accessed on 4 February 2021) to be approved, in progress, or completed. Among all of the studies, 16 have already been completed. Interestingly, most of them (41) are associated with hereditary diseases, mostly retinal diseases (29 out of 56 trials). More precisely, 30.3% (17 out of 56) are related to Leber’s congenital amaurosis, 21.4% (12 of 56) to macular diseases, 19.6% (11 out of 56) to retinitis pigmentosa, 18.0% (10 out of 56) to choroideremia, 9% (5 out of 56) to achromatopsia, and just 1.7% (1 out of 56) are related to corneal diseases (Figure 3C). Considering the types of vectors that are used, adeno-associated virus (AAV) is the most common, confirming that gene replacement therapy is the most widely applied modality in the clinical approach. 

Concerning GTMPs for ocular diseases (Table 3), Vitravene (fomivirsen) was the first GTMP approved by the FDA in 1998, and later by the EMA in 1999. This product, administered by intravitreal injection, was indicated for the treatment of cytomegalovirus (CMV) retinitis in patients with acquired immune deficiency syndrome (AIDS), as these patients are not capable of fighting these infections. The fomivirsen is an antisense phosphorothioate oligodeoxynucleotide complementary to mRNA of the region 2 of human CMV. Hybridization of this antisense molecule to CMV mRNA prevents RNA transcription of the region 2 gene. This inhibits viral replication, a potent antiviral property, and delays the progression of CMV-associated retinitis. Due to commercial motivations, Novartis stopped the US marketing in 2006 and the EU marketing in 2020. However, it is still commercialized in Switzerland. The second authorized GTMP was Macugen (pegaptanib), a product indicated for the wet form of the AMD. It consists of a RNA aptamer that binds to the 165 isoform of vascular endothelial growth factor, producing an anti-angiogenic effect that prevents both the excessive growth of blood vessels and the formation of defective ones [44]. This product was approved by the FDA and EMA in 2006. However, the application was withdrawn in 2011 by Pfizer to include it in the treatment of diabetic macular oedema (Table 3).

Finally, the FDA in 2017 and EMA in 2018 approved Luxturna (voretigene neparvovec), the only GTMP for eye disease currently commercially authorized. This product was designated as orphan medicine by the EMA for the treatment of Leber´s congenital amaurosis in 2012, and for retinitis pigmentosa in 2015, where the RPE65 gene is mutated, producing retinal dystrophy [45]. This product consists of a recombinant AAV vector that delivers a functional RPE65 gene, and it is administered by subretinal injection. Since 2018, this GTMP has been commercialized by Novartis Europharm Limited (Table 3).

### 3.3. Combined ATMPs

As described above and in accordance with Regulation EC No. 1394/2007, cATMPs are composed of a GTMP, sCTMP, or TEP in combination with one or more medical devices or one or more active implantable medical devices as an integral part of the product [1]. Additionally, the biological components of the cATMP must fulfill one of two conditions: “(1) its cellular or tissue part must contain viable cells or tissues, or (2) its cellular or tissue part containing non-viable cells or tissues must be liable to act upon the human body with action that can be considered as primary to that of the devices referred to”. 

To clarify those descriptions, it will be helpful to understand the differences between “medical devices” and “active implantable medical devices”. According to Directive 93/42/EEC, a “medical device” is “any instrument, apparatus, appliance, material or other article whether used alone or in combination, including the software necessary for its proper application intended by the manufacturer, to be used for human beings for the purpose of diagnosis, prevention, monitoring, treatment or alleviation of disease, diagnosis, monitoring, treatment, alleviation of or compensation for an injury or handicap, investigation, replacement or modification of the anatomy or of a physiological process or control of conception, and which does not achieve its principal intended action in or on the human body by pharmacological, immunological or metabolic means” [46]. In contradistinction to a “medical device”, an “active implantable medical device” is “any active medical device (i.e., “any medical device relying for its functioning on a source of electrical energy or any source of power other than that directly generated by the human body or gravity”) which is intended to be totally or partially introduced, surgically or medically, into the human body or by medical intervention into a natural orifice, and which is intended to remain after the procedure” [47]. Both medical device directives are currently under revision to keep up with advances in science and technology, and it is estimated that they will be replaced by new regulations before 2022.

Following these regulations (Directive 93/42/EEC and Directive 90/385/EEC) and the MEDical DEVices guidance document (MEDDEV), a medical device must be approved with the CE marking, an abbreviation in French of “Conformité Européenne” (European Conformity), for it to be commercially available in the EU [46,47,48]. The notified bodies are organizations designated by an EU country to assess the conformity of certain products such as medical devices before being placed on the market. To obtain the CE marking, the manufacturer or marketing company must demonstrate to the notified bodies that their medical device has benefits and an absence of risk. In this regard, any medical device that includes a cATMP must be previously approved with the CE marking by the notified bodies for its commercialization in the EU.

The CAT is the committee responsible for the adoption of scientific recommendations on ATMP classification. Also, for the marketing authorization of a cATMP, the CAT can request the assistance of the notified bodies. Importantly, to qualify as a cATMP, the medicinal product containing a medical device or active implantable medical device must be used in the authorized combination as an integral part of the ATMP. Thus, it must be used for the same purpose as was intended and without additional components [1]. Thus, if the medical device or active implantable medical device is not used, or is no longer used, with the same function as the CE marked medical device, it should be considered as an “excipient” in the final formulation of the drug and not as an integral part. In that case, the ATMP is a non-combined ATMP. 

#### Combined ATMPs for the Eye

cATMPs represent only 1% of the ATMPs that are under development in the EU [8,49]. In 2013, the only cATMP approved in the EU was for the repair of knee cartilage defects. However, it was withdrawn in 2014 for commercial reasons due to the closure of the EU manufacturing site [8,50,51]. To date, 14 clinical trials have been developed (ClinicalTrials.gov, accessed on 4 February 2021) to study ophthalmic applications of a cATMP, NT-501 (Neurotech Pharmaceuticals Inc. (Cumberland, RI, USA) / Enpharma Ltd., Oxford, United Kingdom), to treat retinitis pigmentosa (28.6%; 4 out of 14 trials), macular telangiectasia (21.4%; 3 out of 14 trials), glaucoma (21.4%; 3 out of 14 trials), macular degeneration (14.3%; 2 out of 14 trials), achromatopsia (7.1%; 1 out of 14 trials), and ischemic optic neuropathy (7.1%; 1 out of 14 trials) [51] (Figure 3D). NT-501 consists of encapsulated human retinal pigment epithelial cells genetically modified to secrete therapeutic doses of ciliary neurotrophic factor (CNTF) into the back of the eye (Table 4). The cells are encapsulated by the so-called Encapsulated Cell Technology^®^ (ECT), an intravitreal implant (medical device) that consists of a semi-permeable exterior capsule and an internal scaffolding that allows controlled cell growth [52,53]. The NT-501 implant has demonstrated controlled and continuous release of CNTF at effective doses, with no CNTF or antibodies against CNTF or the cell line in the systemic circulation [51,54,55,56,57,58,59]. This cATMP was designated as orphan drug by the FDA for the treatment of retinitis pigmentosa in 2004 and for treatment of macular telangiectasia type 2 in 2012. Subsequently, the EMA classified NT-501 implant as orphan medicinal product for the treatment of macular telangiectasia type 2 in 2012 and for the treatment of retinitis pigmentosa in 2013 (Table 4) [60,61]. 

## 4. Conclusions

ATMPs provide novel therapeutic approaches to a large variety of diseases and therefore hold the potential to improve the prognosis and even cure certain eye diseases that currently have no effective treatment. However, because of the complexity and novelty of these innovative products, the regulatory procedures have the potential to be excessively rigid and complex, creating new challenges to both developers and regulators. These challenges can be especially significant for small ATMP developers that often have limited budgets and regulatory expertise and for whom a deep understanding and compliance with ATMP regulations can be difficult. It should be noted that, in the EU, most of the ATMP developers are universities, hospitals, charities, and small- and medium-sized enterprises. Therefore, developers normally feel overwhelmed with the regulatory requirements, because they increase both the financial and administrative burden and consequently hamper the market access of ATMP products.

Recently, the development of ATMPs for eye diseases has grown at a very fast and active pace, with many clinical trials conducted worldwide. However, the number of ATMPs that have been approved and that currently have a Marketing Authorization is rather small, as some of them showed poor results and market performance that finally led to their withdrawal. Because the pathway to get the regulatory approval of an ATMP involves a significant amount of expertise, time, and investment, the ATMP classification procedure provided by the CAT is a very helpful tool for developers. The CAT can be consulted to verify whether or not a product based on genes, cells, or tissues is an ATMP, to determine what type of ATMP product it is, and therefore to clarify the development and regulatory path that should be followed. 

In recent years, regulatory agencies from the EU, US, and Japan have implemented schemes such as PRIME (PRIority MEdicines) designation, Breakthrough Therapy designation, and Sakigake designation, respectively, that are accelerating the development of priority cell- and gene-based therapies. To expedite and optimize the approval of ATMPs in these jurisdictions in the near future, a regulatory convergence among these regulatory agencies should be strongly promoted. 

Currently, there are many ongoing clinical trials taking place worldwide for testing the safety and efficacy of new medicinal products based on gene, cells, and tissues for treating ocular conditions. Therefore, if these products provide good results, the number of authorized ATMPs for eye diseases is expected to significantly increase in the coming years. The number of ATMPs under development is growing not only in the regions participating in the International Council for Harmonization of Technical Requirements for Pharmaceuticals for Human Use, but also in all global jurisdictions. As compliance with national and international regulations is of paramount importance in the development of these types of products, stronger efforts should be made to set an international harmonization of the regulatory frameworks that control the development of ATMPs for commercial use. Regulators should be flexible and responsive to the unique and evolving challenges created by ATMPs. Accordingly, scientists and developers must work together with the regulators and act within the requirements of the regulatory system to improve the chances of successfully reaching the market. This will enable patients to have faster access to safe products and get the benefits from them as soon as possible.

## Figures and Tables

**Table 1:** Regulatory framework for cell- and gene-based therapies in the European Union, the United States, and Japan.

Jurisdiction	European Union	United States	Japan
Agency	European Medicines Agency (EMA)	Food and Drug Administration (FDA)	Pharmaceuticals and Medical Devices Agency (PMDA) Ministry of Health, Labour and Welfare (MHLW)
Regulatory framework	Directive 2001/83/EC (related to medical products for human use) European Commission 2007_Regulation EC No. 1394/2007 (related to advanced therapy medicinal products)	Federal Food, Drug, and Cosmetic Act (FDCA) and the Public Health Services Act (PHSA) Regenerative Medicine Advanced Therapy (RMAT) designation: section 3033 of the 21st Century Cures Act.	Act on the Safety of Regenerative Medicine (RM Act) and Pharmaceuticals and Medical Devices Act (PMD Act) 1960 Act No. 145 revised by 2013 Act No. 84
Therapy classification	Somatic cell therapy medicinal products (sCTMPs), tissue engineered products (TEPs), gene therapy medicinal products (GTMPs), and combined ATMPs (cATMPs)	Cell therapy and gene therapy products	Gene-, cell-, and tissue-based therapies

**Table 2:** Somatic cell therapy and tissue engineering medicinal products for the eye.

Product (Commercial Name or Number Designated by EMA ^1^)	sCTMP ^2^ or TEP ^3^	Manufacturer	Active Substance	Administration Route	Indication	Regulatory Status
EU/3/11/874	sCTMP, as implanted cells are expected to help retinal function	Astellas Pharma Europe B.V.(Leiden, The Netherlands)	Human embryonic stem-cell-derived retinal pigment epithelial cells	Intravitreal injection	Stargardt’s disease	Orphan medicinal product designation by the EMA in 2011 Orphan medicinal product designation by the FDA ^4^ for the treatment of Stargardt’s macular dystrophy
EU/3/13/1168	TEP, as implanted cells expected to help corneal regeneration	University of Newcastle. (Newcastle upon Tyne, United Kingdom)	Ex Vivo expanded autologous human corneal epithelium containing stem cells	Transplantation of a cell sheet	Limbal stem cell deficiency	Orphan medicinal product designation by the EMA in 2013
EU/3/14/1340	TEP, as implanted cells, expected to help corneal regeneration	NHS National Services Scotland, trading as Scottish National Blood Transfusion Service. (Edinburgh, United Kingdom)	Culture allogeneic corneal limbal stem cells	Transplantation of a cell sheet	Limbal stem cell deficiency	Orphan medicinal product designation by the EMA in 2014
OraNera (EMEA/H/C/002443)	TEP, as OraNera, expected to replace damaged corneal cells	CellSeed Europe Ltd.. (London, United Kingdom)	Autologous oral mucosal epithelial cells	Transplantation of a cell sheet	Limbal stem cell deficiency	Application for a marketing authorization withdrawn from the EMA in 2013
Holoclar (EU/3/08/579)	TEP (EMA classification)	Holostem Terapie Avanzate S.R.L.(Modena, Italy)	Ex vivo expan ded autologous human corneal epithelium containing stem cells	Transplantation of a cell sheet	Moderate-severe limbal stem cell deficiency, unilateral or bilateral, due to chemical or physical burns	Orphan medicinal product designation by the EMA in 2008 Conditional marketing authorization by the EMA in 2015. The orphan medicinal product designation was maintained
Nepic	Human somatic stem cell-processed products (Japanese PMDA ^5^ classification)	Japan Tissue Engineering Co., Ltd. (Gamagori, Japan)	Human autologous corneal limbus-derived corneal epithelial cell sheet	Transplantation of a cell sheet	Limbal stem cell deficiency	Orphan regenerative medical product designation by the Japanese PMDA in 2020

^1^ EMA, European Medicines Agency. ^2^ sCTMPs, somatic cell therapy medicinal products. ^3^ TEPs, tissue engineered products. ^4^ FDA, Food and Drug Administration. ^5^ PMDA, Pharmaceutical and Medical Devices Agency.

**Table 3:** Gene therapy medicinal products for the eye.

Product (Commercial Name or Number Designated by EMA ^1^)	Manufacturer	Active Substance	Administration Route	Indication	REGULATORY STATUS
Vitravene EMA/H/C/000244	Novartis (Basel, Switzerland)	Fomivirsen (antisense PODN ^2^)	Intravitreal injection	CMV ^3^ retinitis in HIV ^4^ infection	Marketing authorization by the FDA ^5^ 1998 and by the EMA 1999. Withdrawn in 2002 in the EU ^6^ and in 2006 in the US ^7^ Currently authorized in Switzerland
Macugen EMA/671614/2010	Pfizer (New York, USA)	Pegaptanib (RNA aptamer)	Intravitreal injection	Wet form of AMD ^8^/Diabetic macular edema	Marketing authorization by the EMA and by the FDA in 2006/ Withdrawn in 2011 to include a new application (diabetic macular edema)
Luxturna EU/3/15/1518; EU/3/12/981	Novartis	Voretigene neparvovec (AAV ^9^-RPE65)	Subretinal injection	Retinitis pigmentosa/Leber´s congenital amaurosis	Marketing authorization by the EMA in 2018 and by the FDA in 2017

^1^ EMA, European Medicines Agency. ^2^ PODN, phosphorothioate oligodeoxynucleotide. ^3^ CMV, cytomegalovirus. ^4^ HIV, human immunodeficiency virus. ^5^ FDA, Food and Drug Administration. ^6^ EU, European Union. ^7^ US, United States. ^8^ AMD, age-related macular degeneration. ^9^ AAV, adeno-associated virus.

**Table 4:** Combined advanced therapy medicinal product for the eye.

Product (Commercial Name or Number Designated by EMA ^1^)	Manufacturer	Active Substance	Administration Route	Indication	Regulatory Status
NT-501 (EMA/COMP/808529/2012) (EMA/COMP/682942/2012)	Neurotech Pharmaceuticals Inc. (Cumberland, RI, USA) / Enpharma Ltd. (Oxford, United Kingdom)	Encapsulated human retinal pigment epithelial cell line transfected with plasmid vector expressing human CNTF ^3^	Intravitreal implant	Retinitis pigmentosa/Macular telangiectasia type 2	Orphan designation by the FDA ^2^ in 2004 and by the EMA in 2013/Orphan designation by the EMA and the FDA in 2012

^1^ EMA, European Medicines Agency. ^2^ FDA, Food and Drug Administration. ^3^ CNTF, ciliary neurotrophic factor.

## Data Availability

Publicly available datasets were analyzed in this review. This data can be found here: https://www.clinicaltrials.gov/ (accessed on 4 February 2021).
